# Future Trends in Biomaterials and Devices for Cells and Tissues

**DOI:** 10.3390/ijms24043309

**Published:** 2023-02-07

**Authors:** Loredana De Bartolo, Antonella Piscioneri, Seeram Ramakrishna

**Affiliations:** 1 National Research Council of Italy, Institute on Membrane Technology, CNR-ITM, 87036 Rende, Italy; 2 Center for Nanofibers and Nanotechnology, National University of Singapore, Singapore 119077, Singapore

Setting up physiologically relevant in vitro models requires realizing a proper hierarchical cellular structure, wherein the main tissue features are recapitulated. Such a demanding goal necessitates providing cells with a biomimetic surrounding that enables their optimal adaptation in terms of overall cellular performance. Therefore, mimicking the extracellular matrix microenvironment, especially regarding physical and biochemical cues, is a prominent issue for optimal cellular spatiotemporal organization and differentiation. Many innovative approaches in cell culture are used to recapitulate a biomimetic surrounding, including bioreactors, microfluidic devices, biomaterial selection, biomaterial surface functionalization, micro- and nano-engineered scaffolds, responsive biomaterials, electrospinning, cells engineering, and 3D bioprinting. Besides promoting tissue morphogenesis and directing specialized behavior, these systems are reliable platforms for the observed cellular outcomes. For these main features, biomimetic-based systems are ideal for the drug testing/screening of new potential therapeutic molecules and/or for modeling a defined disease in a well-controlled environment. This Special Issue includes original research articles and reviews, providing critical and insightful views of the latest developments. 

The main aspects related to the realization of the 3D bioprinting of cell-laden scaffolds are provided in Zhang et al.’s review [1]. The authors offered an exhaustive discussion on using three-dimensional (3D) bioprinting for human tissue engineering applications. Techniques and approaches are described, from data processing to bioprinting, mainly including inkjet, laser, and extrusion-based techniques. The selection of foundation elements is also reported, enabling readers to easily identify some relevant issues in 3D bioprinting techniques, such as bioink composition and printability. Various bioreactors designed for cell-loaded scaffold maturation are discussed. The dynamic perfusion systems enable the control and monitoring of different parameters (e.g., pH, oxygen, flow rate, temperature), ensuring constant perfusion and waste removal and avoiding mass-transfer limitations of nutrients and oxygen. 

Zare et al.’s review [2] aims to combine state-of-the-art research on pHEMA and its specific applications in relevant fields. The authors deeply discuss the potential of pHEMA for bone tissue regeneration, wound healing, cancer therapy, and ophthalmic uses. The review illustrates how pHEMA is particularly suitable for the healing process thanks to its ability to absorb exudates and promising physicochemical and biological properties. In addition, its transparency allows the constant monitoring of healing progression over time. Special attention is also given to pHEMA use for vision defect repair since this biocompatible polymer is used for contact lenses and ocular drug delivery.

Improved biomaterial biocompatibility is often achieved through different engineered surface strategies. Different surface functionalization approaches are described in a review by Sultana et al. [3], discussing their functional mechanism and application. The most widely used methods are described, including a coating, bioactive coating, plasma spraying coating, and lithography. The review emphasizes the use of emerging surface modification techniques, focusing attention on atomic-scale surface engineering to modify the surface topography in the nanoscale range. The different techniques are illustrated, and their use to enhance biomaterial surface antimicrobial activity is discussed. 

A new stent surface coating constituted by Mg/Al (LDH) nanoparticles loaded with anionic drug biochanin A and further encapsulated with heparin-tagged PLA–PEG copolymer was developed by Adepu et al. [4]. The drug-loaded LDH nanoparticles were encapsulated in PLA–PEG-heparin copolymer to attain a flexible and uniform coating providing a non-thrombogenic and antifouling surface with a controlled drug release. Thus, the developed stent surface coating offers a tunable degradation profile and non-thrombogenicity, preventing restenosis without delaying re-endothelialization.

Lau et al. [5] compared the behavior of HUVEC and human umbilical cord-derived arterial endothelial cells (HUAEC) to establish their potential equivalence for devloping a cardiovascular in vitro model. The most relevant parameters related to endothelial cell state and functionality were investigated and compared between the two types of endothelial cells of different tissue districts. The overall results did not evidence any significant difference between HUVEC and HUAEC, indicating that both cell types are useful cell sources for in vitro endothelial cell research. Furthermore, preclinical results obtained using HUVEC cells reflect the behavior of arterial EC.

Dash et al. [6] propose an interesting paper about altering the metastatic phenotype by inhibiting the nanotubular communication between cancer cells and the endothelium. To this purpose, the authors performed a siRNA-based knockdown of the exocyst complex protein in human cancer cells (MDA-MB-231 cells), which determined a tunneling nanotubes (TNT) formation inhibition. Pharmacological inhibition of the complex Rho GTPases also evoked a consistent reduction of TNT. These observations identified these two protein complexes as potentially responsible for TNT formation. The paper provides relevant insights about targeted mechanisms whose inhibition could reduce the chance of tumor invasion and metastasis. The maintenance of brain slice integrity within an in vitro system is a challenging issue. Herreros et al. [7] developed a brain slice-on-a-chip device for culturing brain slices. The chip device has an innovative injection system that allows the delivery of microvolumes. A test of the injection process, wherein a fluorescent dye was dispensed within the device, allowed the monitoring of the brain slices’ cytoarchitecture. Besides preserving tissue integrity, the in situ delivery system allows for injecting a specific compound within the system and simultaneously visualizing tissue response through imaging analysis. The feasibility of the proposed platform opens its use to other kinds of organotypic cultures. 

Ratlif et al. [8] presented a study aimed at the set-up of an in vitro drug screening platform for glioblastoma treatment. The tumor model is based on patient-derived glioblastoma organoids (PD-GBO), which retain the native tumor features and, therefore, represent a reliable cell source exhibiting real patient tumor characteristics. The organoids, generated from resected tumors, underwent a screening of a panel of 41 FDA-approved drugs. This approach supports personalized patient treatment, offering exhaustive cellular outcomes and molecular profiling in a relevant time frame. 

Overall, this Special Issue provides the latest insights in the regenerative medicine and tissue engineering fields; different approaches and methodologies have the common goal of creating a cell-friendly environment able to evoke optimal cell responses. The manuscripts in this volume also highlight the unmet clinical needs and the future challenges that must be faced for a more personalized path for restoring cellular and tissue functionalities. This knowledge is also helpful to emerging biomedical engineering areas such as smart and intelligent biomaterials, the biomanufacturing of cells and tissues, and personalized medicine.
